# ISCB Honors Temple F. Smith and Eran Segal

**DOI:** 10.1371/journal.pcbi.0030128

**Published:** 2007-06-29

**Authors:** Merry Maisel

## Introduction

Sages have observed that society often honors its living conformists and its dead innovators; “but we prefer to honor living innovators,” says Thomas Lengauer, professor of computational biology, director of the Max Planck Institute for Informatics (Saarbrücken, Germany), and chair of the awards committee of the International Society for Computational Biology (ISCB). The committee, composed of a group of current and past directors of ISCB along with previous award recipients, selects honorees for its two annual awards to innovative scientists. In 2007, the recipients are Temple F. Smith of Boston University (Boston, Massachusetts, United States) and Eran Segal of the Weizmann Institute of Science (Rehovot, Israel). Smith will receive the 2007 ISCB Accomplishment by a Senior Scientist Award, and Segal will receive the 2007 ISCB Overton Prize.

The awards will be presented in Vienna, Austria, at the ISCB's Fifteenth Annual International Conference on Intelligent Systems for Molecular Biology (ISMB), to be held in conjunction with the Sixth European Conference on Computational Biology (ECCB), July 21–25, 2007.

## Accomplishment by a Senior Scientist Award: Temple F. Smith

**Image 1:**
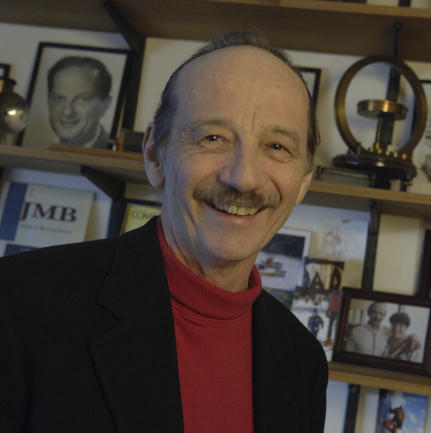
Temple F. Smith (Credit: Kalman Zabarsky, Boston University)

The BioMolecular Engineering Research Center (BMERC) at Boston University, directed by Professor Temple F. Smith (see Image 1), has two major research objectives. The first is to develop statistical and other computational approaches to the detection of syntactic and semantic patterns in DNA, RNA, and protein sequences. The second is to use the approaches thus developed to identify structure, function, and regulation in DNA, RNA, and proteins.

The pursuit of these objectives, Smith notes, “has led to the formulation and testing of major hypotheses in the areas of molecular evolution, gene regulation, developmental genetics, and protein structure–function relationships.” The success of Smith's research program has been attested to in many ways, not least by his receipt of the ISCB's Accomplishment by a Senior Scientist Award for 2007.

The award recognizes members of the computational biology community who have made major contributions to the field through research, education, service, or a combination of the three. It acknowledges community members who are more than 12 years post-degree.

“Professor Smith's contributions go well beyond those for which he is best known,” says Lengauer, “and he is a towering figure in bioinformatics, one of the founders of the discipline. In addition to starting GenBank and being the Smith of the Smith–Waterman algorithm, he has done seminal work on the entropy of the genetic code and on pattern-directed protein structure prediction. Other influential work includes research on gene prediction, molecular phylogenies, multiple sequence alignments, and the analysis of sequence patterns. His results have had tremendous impact on the field. He has been an organizer and co-organizer of the meetings and symposia of the multiple disciplines that intersect in computational biology as well as of educational and outreach programs for young students. Smith feels that the organizing of the ‘Waterville Valley' meetings [starting in 1986] was key in introducing many younger scientists to both bioinformatics and the fields of the older leaders. He has served on undergraduate, graduate, curriculum, and tenure committees as well. And his BioMolecular Engineering Research Center at the Boston University College of Engineering is a superlative resource for a wide variety of endeavors.”

Smith obtained his doctorate in nuclear physics from the University of Colorado in 1969 and was a National Institutes of Health (NIH) postdoctoral fellow with Stanislaw Ulam, T. T. Puck, and John R. Sadler, studying bacterial genetic regulation. He then took an appointment as professor of physics at Northern Michigan University, spending summers as a visiting staff member in applied mathematics and theoretical biology at Los Alamos Scientific Laboratory, with Stanislaw Ulam, George Bell, Walter Goad, Bill Beyer, and, of course, Mike Waterman. It was there that he helped to organize GenBank.

In 1978–1979, Smith spent a sabbatical year working with Harold Morowitz at Yale University, exploring the relationships among biology, physics, and history. In the following year, he was a visiting professor at the University of Southern California. From 1985 to 1991, Smith directed the Molecular Biology Computational Research Resource of Dana-Farber Cancer Institute, Harvard Medical School, and Harvard School of Public Health.

Moving to Boston University in 1991, Smith became a professor in the departments of bioengineering and pharmacology and director of BMERC. His center is currently working under NIH and NSF (National Science Foundation) grants on activation of inflammation stress response pathways, cellular signaling problems (with the Alliance for Cellular Signaling), the generation of automated models of protein folds, and the core genomics of the origin of eukaryotes. Smith serves on the editorial boards of *Molecular Biology and Evolution* and the *Journal of Computational Biology.*

Interviewed recently by a science writer at Boston University, Smith explained how he and Waterman came to write their only geology paper. It was a serendipitous event, Smith told Michael Seele, that occurred when Waterman visited him at Yale University. Seele wrote, for an upcoming issue of the *Boston University College of Engineering Magazine*:

“As the pair walked to lunch, they passed through the geology department lobby, where two large core samples on display stopped them in their tracks. Similar sequences of strata on different columns were connected by strings. Smith and Waterman immediately saw the columns as strands of DNA and the comparable strata as the short protein sequences they were trying to align. ‘We now faced the possibility that a geologist had solved the problem before us,' Smith said. Resigned, Smith and Waterman visited the geology chairman and asked how the sequence alignment had been done. Their mood elevated when the chairman informed them that visual observation and string were as far as anyone had advanced with a solution. ‘Lo and behold! This was an unsolved problem in geology,' Smith said. ‘This resulted in our first geology paper, basically written over the next couple of days.' With a fresh perspective, the team returned to bioinformatics work and published the Smith–Waterman sequence alignment algorithm the following year. It remains one of the most referenced papers in molecular biology.”

Of his award, Smith says, “I'm truly honored to join my longtime friend and colleague Mike Waterman, who preceded me in winning this award last year, as well as the distinguished company of previous winners.

“People sometimes date the history of our field back to the algorithm that I wrote with Mike. I find, instead, that the seeds of both computational approaches and general interdisciplinarity were sown somewhat earlier—for example, at a Symposium on Evolving Genes and Proteins held at Rutgers [University] in 1964 and published in *The Quarterly Review of Biology* in March 1966. The problems of our age are also prefigured in the works of those we count as the great thinkers in evolutionary biology: J. B. S. Haldane, D'Arcy Thompson, Sewall Wright, Salvador Luria, and Max Delbrück. As I hope to explain in my keynote address at ISMB/ECCB 2007, what is new today in computational biology is the wealth of data that we have and the overwhelming complexity of biological systems as we move toward future challenges in bioinformatics.”

Last year's winner of the Accomplishment by a Senior Scientist Award, Mike Waterman, will present the award in Vienna and introduce Temple Smith's keynote address, titled “Computational Biology: What's Next?” to close the conference on July 25, 2007. To read additional biographical information and an abstract of Smith's keynote address, see http://www.iscb.org/ismbeccb2007/keynotespresentations/#smith.

## Overton Prize: Eran Segal

As a freshman biology student should learn, every eukaryotic organism's genome, carried in every somatic cell nucleus, is packaged into a structure called chromatin. The basic repeating unit of chromatin is the nucleosome, and each nucleosome consists of histone proteins around which 147 basepairs of the genomic DNA are wrapped. To accommodate a genome of 10 million basepairs, the chromatin is likely to contain about 50,000 nucleosomes. The largest genomes are estimated to contain up to 100 billion basepairs, and the chromatin to package that would need to have 500 million nucleosomes, all in the nuclear space of a few microns in diameter.

**Image 2:**
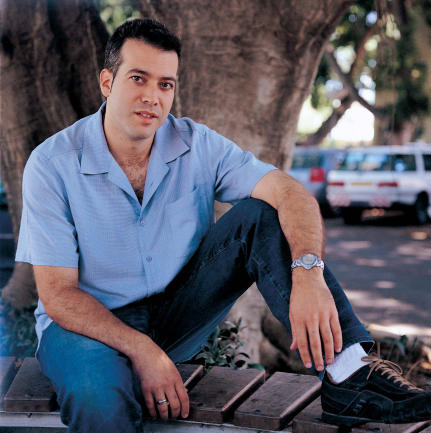
Eran Segal (Credit: Weizmann Institute of Science)

But what determines the detailed positions of nucleosomes along the genome? Such positions are important for even the most basic cellular processes, including transcription, since these processes are carried out by the binding of regulatory factors to “naked” DNA basepairs that are not within nucleosomes. Scientists have agreed that there must be some nonrandom control at work, but there has been a lot of argument about just what it might be. In the summer of 2006, Eran Segal (see Image 2) of the Weizmann Institute of Science (Rehovot, Israel) and colleagues published a study in *Nature* (442: 772–778) hypothesizing that the instructions for wrapping DNA around nucleosomes are contained in the DNA itself. The authors used a statistical computational model to predict exactly how that is done, and they completed the proof by verifying the predictions with experiments in yeast.

“This important paper brought Segal and his main collaborator, experimentalist Jonathan Widom of Northwestern University, a lot of attention,” says Lengauer. “It was featured in *Nature's* ‘News and Views' section in an article by Tim Richmond, and the work was also described in a ‘Making the Paper' section. And it made *The New York Times* on July 25. But those of us who attend the ISMB and RECOMB [Research in Computational Molecular Biology] conferences have been aware of Eran Segal for a lot longer. Papers written for his doctoral thesis have won best paper and best student paper awards at both meetings. He has introduced very novel ways of using statistical learning methods to uncover important aspects of the regulatory program that is running in cells.”

ISCB established the Overton Prize in 2001 in memory of G. Christian Overton, who was director of the Center for Bioinformatics at the University of Pennsylvania and a major contributor to the field. “He was a member of the ISCB Board of Directors, and his sudden death in 2000 was a shock to the community,” Lengauer said. “Those of us who remember Chris Overton remember the kind of work he did—however laborious it was, it was always exciting and thought-provoking, dominated by an innovative spark. Eran Segal seems to me to be especially deserving of this award in Chris's memory.”

Segal obtained his B.Sc. (summa cum laude) in computer science from Tel Aviv University in 1998. He did his doctoral work in computer science and genetics at Stanford University, obtaining his Ph.D. in 2004. His advisor, Daphne Koller, remembers him vividly. “One of Eran's most impressive qualities,” she says, “is his ability to get things done effectively and extremely well. He would be working on five projects, and I would be sure that at most one would get done. But not with Eran—he just kept producing idea after idea, result after result, paper after paper. Part of it was the long hours, but a larger part was that he is an extremely talented and effective researcher.

“Another of his important qualities,” she continues, “is his ability to produce meaningful innovations in two distinct disciplines. On the one side, he identifies real biological problems, which biologists care about; on the other, he comes up with novel computational solutions that are well-founded and technically insightful.”

Segal spent a year as a research fellow at the Center for Physics and Biology at Rockefeller University before joining the Weizmann Institute of Science in 2005. “My lab develops quantitative statistical models aimed at understanding how molecular components interact in performing complex biological functions,” Segal says. “We are interested in the control of transcription and translation and the structure of chromatin as it contributes to these. We are currently applying our ideas to the transcriptional network of the *Drosophila* embryo in an attempt to develop thermodynamic models that will explain how cells compute the expression patterns of the system from the *cis*-regulatory DNA sequence and binding-site preferences of the participating transcription factors. We're also continuing our work on the DNA sequence preferences of nucleosomes and the way in which they specify the overall nucleosome organization.”

Of his award, Segal says, “I'm very much honored to be singled out, and I must thank my mentors, Daphne Koller and Nir Friedman, and my students, colleagues, and collaborators, people without whose efforts no progress could be made. In particular, I am enjoying close collaborations with several experimentalists such as Jon Widom, Ulrike Gaul, and Howard Chang, and I'm extremely appreciative of their ability to confirm or refute in vivo the results that emerge from our lab's efforts in silico. We, in turn, take cues from their results in revising or adjusting our models. This prize affirms the value of our process.”

Eran Segal will be given the 2007 ISCB Overton Prize in Vienna at ISMB/ECCB 2007, and he will give a keynote address on July 23. To read additional biographical information and view an abstract of his talk, “Quantitative Models for Chromatin and Transcription Regulation,” see http://www.iscb.org/ismbeccb2007/keynotespresentations/#segal.

## Additional Information

For the full agenda and registration information for ISMB/ECCB 2007, where these ISCB award winners will speak, and where there will be eight other keynote lectures plus nearly 200 additional scientific talks, please visit the conference Web site at http://www.iscb.org/ismbeccb2007. For a review of past ISCB Accomplishment by a Senior Scientist Award and Overton Prize winners, please see http://www.iscb.org/ssaa.shtml and http://www.iscb.org/overton.shtml, respectively.

